# Increase in serum brain-derived neurotrophic factor levels during early withdrawal in severe alcohol users

**DOI:** 10.47626/2237-6089-2021-0254

**Published:** 2022-10-18

**Authors:** Andrei Garziera Valerio, Felipe Ornell, Vinicius Serafini Roglio, Juliana Nichterwitz Scherer, Jaqueline Bohrer Schuch, Giovana Bristot, Flavio Pechansky, Flavio Kapczinski, Felix Henrique Paim Kessler, Lisia von Diemen

**Affiliations:** 1 Centro de Pesquisa em Álcool e Drogas Hospital de Clínicas de Porto Alegre Universidade Federal do Rio Grande do Sul Porto Alegre RS Brazil Centro de Pesquisa em Álcool e Drogas , Hospital de Clínicas de Porto Alegre , Universidade Federal do Rio Grande do Sul (UFRGS), Porto Alegre , RS , Brazil .; 2 Programa de Pós-Graduação em Psiquiatria e Ciências do Comportamento UFRGS Porto Alegre RS Brazil Programa de Pós-Graduação em Psiquiatria e Ciências do Comportamento , UFRGS , Porto Alegre , RS , Brazil .; 3 Programa de Pós-Graduação em Saúde Coletiva Universidade do Vale do Rio dos Sinos São Leopoldo RS Brazil Programa de Pós-Graduação em Saúde Coletiva , Universidade do Vale do Rio dos Sinos (Unisinos), São Leopoldo , RS , Brazil .; 4 Laboratório de Psiquiatria Molecular Hospital de Clínicas de Porto Alegre Porto Alegre RS Brazil Laboratório de Psiquiatria Molecular , Hospital de Clínicas de Porto Alegre (HCPA), Porto Alegre , RS , Brazil .; 5 Programa de Pós-Graduação em Bioquímica UFRGS Porto Alegre RS Brazil Programa de Pós-Graduação em Bioquímica , UFRGS , Porto Alegre , RS , Brazil .; 6 Department of Psychiatry and Behavioural Neurosciences McMaster University and St. Joseph’s Healthcare Hamilton Hamilton ON Canada Department of Psychiatry and Behavioural Neurosciences , McMaster University and St. Joseph’s Healthcare Hamilton , Hamilton , ON , Canada .

**Keywords:** BDNF, alcohol dependence, addiction, neurotrophin, abstinence

## Abstract

**Introduction:**

Changes in brain-derived neurotrophic factor (BDNF) have been linked to the neuroadaptative consequences of chronic alcohol use and associated with disease severity and prognosis. Few studies have evaluated the influence of drug withdrawal and clinical and sociodemographic data on BDNF levels in severe alcohol users.

**Objectives:**

Our goals were (1) to evaluate variation in BDNF levels during alcohol withdrawal and, (2) to assess the influence of putative confounding factors on BDNF levels.

**Methods:**

Our sample consists of 62 men with alcohol use disorder undergoing a detoxification process. Serum BDNF levels were measured using a commercial sandwich-ELISA kit, at two points: before and after the detoxification period.

**Results:**

We found an increase in BDNF levels during alcohol withdrawal (25.4±9.6 at admission vs. 29.8±10.2 ng/ml at discharge; p < 0.001), even after controlling for potential confounders (positive family history, number of days between blood sample collections, and age) (Generalized Estimating Equation: coefficient = -4.37, 95% confidence interval [95%CI] -6.3; -2.4; p < 0.001). Moreover, individuals who had first-degree relative with alcohol dependence had smaller increases in BDNF levels than individuals with no family history (14.8 [95%CI -5.3; 35.6] vs. 35.3 [95%CI 15.4; 74.8]; p = 0.005).

**Conclusions:**

In summary, variation in BDNF levels seems to be influenced by withdrawal in severe alcohol users. A positive family history of alcohol dependence could also be a factor that influences variation in this biomarker.

## Introduction

The pathogenesis of substance use disorder (SUD) involves many biological mechanisms and neuroadaptive changes, with notable involvement of neurotrophins. Brain-derived neurotrophic factor (BDNF) is the most abundant neurotrophin in the human brain and is associated with neurogenesis, cognitive functions, cerebral neuroplasticity, learning, and memory. ^1 - 3^ Consistent evidence shows changes in BDNF regulation underlying several behaviors and psychiatric disorders. ^4^ In fact, changes to serum and plasma BDNF levels were observed in individuals with SUD. ^5^ Moreover, it has been shown that severity of drug abuse was inversely correlated to BDNF levels, ^6 - 9^ suggesting that BDNF could be a prognostic marker in SUD. ^10 - 12^

Alcohol use disorder is the most prevalent SUD, with a prevalence of 5.1% among adults, affecting approximately 283 million people worldwide. ^13^ Lower levels of BDNF have been observed among current alcohol users, but studies are still inconsistent and controversial, depending on the characteristics of the samples. ^11 , 14 - 16^ During the withdrawal phase, some studies detected a small increase in serum BDNF levels, ^9 , 17^ while others studies show decreases in this neurotrophin during the first days of alcohol abstinence. ^18 , 19^ In addition, lower levels of BDNF were found in individuals with delirium tremens (DT), even after detoxification. ^7^ A follow-up study showed that individuals who were abstinent for 180 days had higher levels of serum BDNF compared to baseline measures and compared to those who relapsed during this same period. ^11^

Beyond use of drugs, other factors may also be related to the BDNF variation during alcohol abstinence and could be influencing the results detected so far, including age, sex, and age at first drug use, ^5^ presence of psychiatric disorders, ^20 , 21^ neurodegenerative diseases, chronic inflammatory state, ^22^ tobacco consumption, ^23^ family history of alcohol, ^24 , 25^ and genetic predisposition. ^26 , 27^ Currently, there are no biomarkers that can predict the overall severity or disease stage in SUD, although assessment of peripheral biomarkers in specific populations might shed light on the relationship between such markers, including BDNF, and clinical characteristics and disease progression.

The overall scenario suggests that BDNF could be a candidate biomarker of severity and prognosis in alcohol addiction. Nonetheless, only a few studies have evaluated BDNF levels in severe alcohol users during early withdrawal. In this sense, our main goal was to evaluate the variation of BDNF levels before and after alcohol withdrawal in individuals with alcohol use disorder during an inpatient treatment program. The influence of putative confounding factors on BDNF levels during alcohol withdrawal was also assessed.

## Methods

### Sample selection

Alcohol users were recruited at the Álvaro Alvim Unit, a specialized service for the treatment of addiction in male patients at the Hospital de Clínicas de Porto Alegre (HCPA), a public hospital located in Southern Brazil. The study was approved by the HCPA Institutional Review Boards and Ethics Committees (Number 14-0249), and all subjects enrolled provided written informed consent.

Inclusion criteria were: (1) a diagnosis of alcohol use disorder according to the criteria from the fourth version of the Diagnostic and Statistical Manual of Mental Disorders (DSM-IV); (2) age 18 years or older; and (3) consent to provide two blood samples during inpatient treatment. Individuals were excluded if they were unable to participate or understand the research protocol, judged on clinical status. Individuals were recruited consecutively between October 2013 and May 2016, during which time all patients admitted were invited to participate in the study.

The research protocol was applied by trained junior researchers, after initial detoxification and stabilization of withdrawal symptoms. Sociodemographic data and psychiatric disorders respectively were assessed using the Addiction Severity Index – 6th Version (ASI-6), previously validated for Brazilian Portuguese, ^28^ and the Structured Clinical Interview for DSM-IV. These interviews were conducted between the fifth and 12th days in hospital. Initially, 94 inpatients agreed to participate in the study. However, only 62 individuals completed the research protocol and provided two blood samples (one at hospital admission and another one while in hospital) and were therefore included in the study.

### Blood collection and processing

Two blood samples were collected. The first blood sample was collected within the initial 24 hours after admission and the second was taken after 15 days in hospital. For both samples, ten milliliters of blood were collected from each patient after 8h fasting by venipuncture into an anticoagulant-free vacuum tube. Immediately after collection, blood samples were centrifuged at 4000rpm for 10 min and the serum was aliquoted, labeled, and stored at -80ºC until assay testing.

### BDNF measurement

Serum BDNF levels were measured by sandwich-ELISA using a commercial kit, according to the manufacturer’s instructions (Millipore, USA). Briefly, microtiter plates (96-well, flat-bottom) were incubated overnight at 4ºC with the samples diluted 1:75 in sample diluent and standard curve ranging from 15.63 to 1000 pg/mL of BDNF. Plates were washed four times with wash buffer followed by addition of biotinylated mouse anti-human BDNF monoclonal antibody (diluted 1:1000 in sample diluent), which was incubated for 3 hours at room temperature. After washing, samples were incubated with streptavidin-horseradish peroxidase conjugate solution (diluted 1:1000 in sample diluent) for 1 hour at room temperature. After addition of the substrate and stop solution, the amount of BDNF was determined (absorbance set at 450 nm). The standard curve demonstrates a direct relation between optical density and BDNF concentration.

### Statistical analysis

Distributions of continuous data were assessed using the Shapiro-Wilk test. Variables with normal distribution were expressed as mean and standard deviation, while other variables were expressed as median and 1st-3rd quartile (IQR). Categorical variables were expressed as absolute and relative frequency.

Two different measures of BDNF levels were obtained: at hospital admission and after 15 days in hospital (named “BDNF discharge”). Initially, the mean difference between these two measures was assessed using the paired *t* test. For subsequent analyses, BDNF levels were transformed into a single measure that considers the percentage variation in levels, using the following formula:


$$\left[\left({\text{BDNF}}_{\text{discharge}}-{\text{BDNF}}_{\text{admission}}\right)/{\text{BDNF}}_{\text{admission}}\right]\times 100$$

Bivariate analyses were conducted to assess the relationships between the percentage variation in BDNF levels and continuous data (i.e.: age, years of regular use of alcohol) or categorical data (i.e.: presence of psychiatric disorders, family history) using Spearman’s coefficient or the Mann-Whitney test, respectively. Moreover, a generalized estimating equation (GEE) model was run to analyze serum BDNF levels at admission and discharge, controlling for potentially confounding variables: (1) presence of first-degree relatives with alcohol dependence (yes/no); (2) number of days between the two blood collections; and (3) age.

## Results

### Demographic characteristics and psychiatric diagnoses

The sample consisted mostly of white men (n = 46, 74.2%), with mean age of 48.9 (SD = 9.2) years, lower education level (59.7% with less than 8 years’ schooling), and currently living without a partner (64.5%). Patients had high prevalence of current anxiety symptoms (29.4%) and major depressive episodes (19.6%). At least two previous treatments for problems with alcohol were reported by 50% of the sample, and almost 76% had a positive family history of alcohol use disorder. Also, half of the sample had consumed alcohol three or more times per week for more than 22 years ( Table 1 ).

**Table 1:** Sociodemographic and clinical data and percentage variation of BDNF between admission and discharge

	Total (n = 62)	BDNF %var	p-value
Age at first use of alcohol*	15.4±4.0	0.171	0.184
Years of regular use of alcohol (3+ times/week)* (n = 58)	22 [10; 30]	-0.117	0.700
Number of hospitalizations for alcohol use*	2 [0; 5]	0.038	0.778
Age (years)*	48.9±9.2	0.018	0.887
BMI*	25.8±4.1	0.033	0.798
Skin color ^†^			0.664
White	46 (74.2)	16.7 [-2.2; 37.5]	
Non-white	16 (25.8)	19.8 [6.2; 42.7]	
Educational level ^†^			0.971
≤ 8 years of schooling	37 (59.7)	15.2 [5; 38.7]	
> 8 years of schooling	25 (40.3)	24.3 [-2.2; 38.5]	
Marital status ^†^			0.871
Not married	40 (64.5)	16.2 [3.6; 36.6]	
Married	22 (35.5)	19.8 [-1; 41.4]	
Homeless ^†^ (n = 49 ^‡^ )			0.834
Yes	7 (14.3)	15 [3.6; 43.1]	
No	42 (85.7)	16.2 [-1; 38.7]	
First-degree relative with alcohol use disorder ^†^			**0.005**
Yes	47 (75.8)	14.8 [-5.3; 35.6]	
No	15 (24.2)	35.3 [15.4; 74.8]	
Major depressive episode ^†^ (n = 51)			0.367
Current presence	10 (19.6)	13.3 [-6.5; 34.1]	
Absence	41 (80.4)	18.8 [5; 41.4]	
Anxiety disorders ^†^ (n = 51)			0.352
Current presence	15 (29.4)	24.3 [3.7; 71.7]	
Absence	36 (70.6)	15.3 [1.3; 38.6]	
Smoking (current) ^†^			0.249
Yes	41 (66.1)	17.1 [-1.0; 34.1]	
No	21 (33.9)	15.4 [5.0; 74.9]	
Chronic diseases§			0.249
Yes	16 (27.6)	40.2 [0.3; 74.3]	
No	42 (72.4)	14.9 [3.6; 32.6]	

Categorical data were expressed as n (%), and continuous data as mean ± standard deviation or median [1st; 3rd quartiles].

* Spearman correlations; ^†^ Mann-Whitney test.

^‡^ Has been homeless at some time in life.

^**§**^ At least one of the following diseases: diabetes, cancer, HIV, stroke, or liver disease.

### BDNF levels

**Figure 1:**
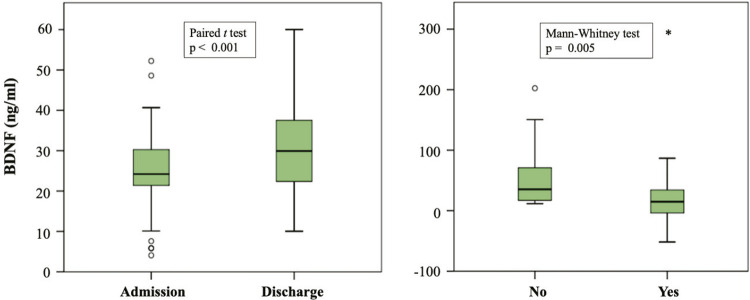
Variation in BDNF levels between hospital admission and discharge (n = 62). A) BDNF levels after alcohol withdrawal (paired *t* test). B) Percentage variation in BDNF and first-degree relatives with alcohol dependence (no relative (n = 15), has relative (n = 47); Mann-Whitney test).

The comparison between admission and discharge measurements showed an increase in BDNF levels after alcohol withdrawal (25.4±9.6 vs. 29.8±10.2 ng/mL; p < 0.001, Figure 1A ). Furthermore, analyses considering sociodemographic and clinical data demonstrated that the percentage of variation in BDNF levels was significantly lower for those who had a first-degree relative with alcohol dependence (14.8 [-5.3;35.6] vs. 35.3 [15.4;74.8]; p = 0.005, Table 1 and Figure 1B ). No other associations were found with sociodemographic or clinical characteristics or psychiatric diagnoses ( Table 1 ). Liver function tests (alanine transaminase, aspartate transaminase, and gamma-glutamyltransferase) were also unrelated to BDNF variation (Table S1, available as online-only supplementary material).

Taking into account these results, and to confirm our initial finding, the comparison between BDNF levels at admission and discharge was assessed controlling for presence of first-degree relatives with alcohol dependence and also considering the number of days between blood collections and age as possible confounding variables. The increase in BDNF levels during withdrawal remained significant (coef. = -4.37, 95% confidence interval [95%CI] -6.3; -2.4; p < 0.001, Table 2 ).

**Table 2:** Generalized estimating equation (GEE) model for change in BDNF levels between hospital admission and discharge

	Coef.	95%CI	p-value
Age	-0.126	(-0.4; 0.2)	0.405
Admission (discharge ref.)	-4.367	(-6.3; -2.4)	< 0.001
First-degree relatives with alcohol dependence	0.112	(-5.9; 6.1)	0.971
Days between blood sample collections	0.003	(-0.1; 0.1)	0.964

95%CI = 95% confidence interval.

## Discussion

Our main finding involves an increase of BDNF levels during early withdrawal in severe alcohol users. These results are in line with previous studies conducted with alcohol users. ^7 , 11^ Of note, our results also suggest that the presence of family history of alcohol use disorder contributes to the variation in BDNF levels during abstinence, perhaps due to genetic influence.

Increased BDNF levels during abstinence have been observed in alcohol addiction, although some aspects may differ between studies. For instance, different abstinence periods can be assessed and considered. Our study observed that the variation in BDNF levels occurs shortly after alcohol withdrawal (on average 15 days later). Sönmez et al. ^29^ also assessed BDNF levels 2 weeks after alcohol withdrawal and suggested that BDNF could be involved in neuroadaptation during abstinence. Similar evidence has been observed in relation to other drugs, like crack cocaine ^6 , 30 - 32^ and heroin. ^33^

Furthermore, preclinical studies indicate that BDNF levels appear to vary according to the pattern of alcohol consumption, whether recreational use, abuse, or dependence. ^34 , 35^ Acute and moderate use of alcohol temporarily increases BDNF levels, while chronic and excessive use seems to lead to a reduction in levels. ^34 , 36^ The withdrawal period appears to bring BDNF back to baseline levels, ^19 , 34 , 36 - 38^ which corroborates our results, since our sample includes chronic and severe alcohol users. In fact, the severity of abstinence may also influence BDNF levels. ^7^ Patients with DT have lower levels of BDNF compared to healthy controls and patients without DT. After detoxification, BDNF levels increase in alcoholic patients, but to a lesser extent in those with DT. ^7 , 39^

This pattern of BDNF levels during withdrawal may be related to the brain’s capacity to regenerate after discontinuation of substance use. ^40^ Chronic use of psychoactive substances involves repeated hyperactivation of the dopaminergic pathway, leading to neuroadaptive mechanisms that can cause disruption in the brain’s reward system and in regulation of BDNF. ^41 - 44^ Although, this reorganization may indicate a functional response mechanism in the short term, over the long term, when related to chronic use of alcohol for many years, it can generate dysfunctional changes (allostatic load), resulting in a harmful response. ^45 - 51^ It is suggested that in advanced stages of the disorder, inadequate responses may persist even after abstinence. ^52 , 53^

This phenomenon is encompassed by the term neuroprogression, which is related to pathological reorganization of the central nervous system (CNS) along the course of severe psychiatric mental disorders. Understanding of the biological underpinnings of neuroprogression is still recent. ^54^ In alcohol addiction, as well as in other psychiatric disorders, it is believed that homeostatic functioning is disturbed over the course of the pathology by remodeling of the CNS. ^55 - 57^ Investigations that have evaluated BDNF in other psychiatric disorders, such as schizophrenia, major depressive disorder, bipolar disorder, and suicide behavior, also showed a similar pattern to that detected in our analyses. ^58 - 65^ Although presence of psychiatric disorders could lead to changes in BDNF levels, no influence associated with such comorbidities was observed in our study. The concentration and function of BDNF might also be influenced by chronic conditions such as diabetes, ^66^ cancer, ^67^ HIV, ^68^ stroke. ^69^ and liver disease. ^70^ Nonetheless, no association was found between presence of these diseases and variation in BDNF levels in our sample, emphasizing that the increase in BDNF levels is mainly due to alcohol withdrawal.

This study has some limitations. Previous research has shown that sex-related hormonal, genetic, and epigenetic factors can modulate BDNF activity. ^71 - 74^ However, this study was carried out at an exclusively male psychiatric hospital and therefore does not allow us to evaluate the effect of variables related to sex. Some other factors regarding the hospitalization process, such as use of medication, ^75^ may have also contributed to the variation in BDNF levels. However, during the initial period of abstinence, patients are only given medications to manage withdrawal symptoms (such as benzodiazepines). Other medications (such as antidepressants and mood stabilizers) are usually prescribed after this initial period. Other non-pharmacological measures, such as group therapy sessions ^76^ and physical exercises ^76 - 78^ were also very similar among all patients. These factors may not therefore have an influence on our findings. On the other hand, individual variations related to clinical improvement and presence of withdrawal symptoms may also impact BDNF levels. Although all patients included in the study exhibited clinical improvement during follow-up, we did not apply any scale that specifically assesses the progression of withdrawal symptoms. Also, the sample size is small, which may have prevented us from detecting other significant findings. Nonetheless, it should be noted that this is a more homogeneous sample since all patients are men, who were admitted to hospital for treatment of severe cases of addiction, were refractory to outpatient treatment, and were possibly at a more advanced stage of the condition.

## Conclusions

Our findings reinforce the role of BDNF as a neurotrophin involved in alcohol use disorder. The variation in BDNF levels during alcohol withdrawal reinforces the hypothesis that BNDF is a possible biomarker of this pathology. Also, our study identified that presence of family history of alcohol use disorder could be a factor that influences the variation in this biomarker. Further studies are needed to understand the relationship between BDNF, severity (or staging), and prognosis in alcohol use disorders, since this topic is of pivotal importance for clinical practice as well as for scientific research.

## Supplementary material


