# Supplementary material for: Increase in serum brain-derived neurotrophic factor levels during early withdrawal in severe alcohol users
Source: Trends Psychiatry Psychother. 2022 Oct 18;44:e20210254. doi: 10.47626/2237-6089-2021-0254 (PMC10039723; doi:10.47626/2237-6089-2021-0254)
Supplement: Supplementary file 1 [file 2238-0019-trends-44-e20210254_suppl01.pdf]

http://doi.org/10.47626/2237-6089-2021-0254

Table S1 - Liver function tests

	Total (n = 62)	BDNF%var	p-value
Alanine transaminase	30 [18; 53]	0.062	0.631
Aspartate transaminase	29 [20; 61]	0.076	0.556
Gamma-glutamyltransferase*	65 [40; 123]	0.131	0.342

Data presented as Median [1st; 3rd quartile].

Spearman correlation with BDNF percentage variation.

*n = 55.
